# Surface Modification of Biodegradable Polymers towards Better Biocompatibility and Lower Thrombogenicity

**DOI:** 10.1371/journal.pone.0142075

**Published:** 2015-12-07

**Authors:** Andreas Rudolph, Michael Teske, Sabine Illner, Volker Kiefel, Katrin Sternberg, Niels Grabow, Andreas Wree, Marina Hovakimyan

**Affiliations:** 1 Institute for Biomedical Engineering, Rostock University Medical Center, Friedrich-Barnewitz-Strasse 4, D-18119, Rostock, Germany; 2 Department of Transfusion Medicine, Rostock University Medical Center, Ernst-Heydemann-Strasse 6, D-18057, Rostock, Germany; 3 Institute of Anatomy, Rostock University Medical Center, Gertrudenstrasse 9, D-18057, Rostock, Germany; North Carolina A&T State University, UNITED STATES

## Abstract

**Purpose:**

Drug-eluting stents (DES) based on permanent polymeric coating matrices have been introduced to overcome the in stent restenosis associated with bare metal stents (BMS). A further step was the development of DES with biodegradable polymeric coatings to address the risk of thrombosis associated with first-generation DES. In this study we evaluate the biocompatibility of biodegradable polymer materials for their potential use as coating matrices for DES or as materials for fully bioabsorbable vascular stents.

**Materials and Methods:**

Five different polymers, poly(L-lactide) PLLA, poly(D,L-lactide) PDLLA, poly(L-lactide-co-glycolide) P(LLA-co-GA), poly(D,L-lactide-co-glycolide) P(DLLA-co-GA) and poly(L-lactide-co-ε-caprolactone), P(LLA-co-CL) were examined *in vitro* without and with surface modification. The surface modification of polymers was performed by means of wet-chemical (NaOH and ethylenediamine (EDA)) and plasma-chemical (O_2_ and NH_3_) processes. The biocompatibility studies were performed on three different cell types: immortalized mouse fibroblasts (cell line L929), human coronary artery endothelial cells (HCAEC) and human umbilical vein endothelial cells (HUVEC). The biocompatibility was examined quantitatively using *in vitro* cytotoxicity assay. Cells were investigated immunocytochemically for expression of specific markers, and morphology was visualized using confocal laser scanning (CLSM) and scanning electron (SEM) microscopy. Additionally, polymer surfaces were examined for their thrombogenicity using an established hemocompatibility test.

**Results:**

Both endothelial cell types exhibited poor viability and adhesion on all five unmodified polymer surfaces. The biocompatibility of the polymers could be influenced positively by surface modifications. In particular, a reproducible effect was observed for NH_3_-plasma treatment, which enhanced the cell viability, adhesion and morphology on all five polymeric surfaces.

**Conclusion:**

Surface modification of polymers can provide a useful approach to enhance their biocompatibility. For clinical application, attempts should be made to stabilize the plasma modification and use it for coupling of biomolecules to accelerate the re-endothelialization of stent surfaces *in vivo*.

## Introduction

Cardiovascular diseases (CVD), including a spectrum of pathologies, continue to be the leading cause of mortality, accounting for approximately 30% of deaths worldwide [1–3]. In 2012, about 17 million people died on CVD, and this number is expected to rise to more than 23.6 million by 2030 [4,5]. Among all CVD, the coronary artery disease (CAD) accounts for approximately one-third to one-half of the total cases [6]. The standard interventional options for revascularization in CAD include balloon angioplasty alone or followed by stent deployment, unless blockage is so severe, that bypass grafting becomes necessary.

The introduction of drug eluting stents (DES), which allow the delivery of drugs in a controlled manner to the arterial wall significantly reduced the rate of restenosis, occurring after bare metal stent (BMS) implantation [7,8]. Restenosis caused by neointimal hyperplasia is a side-effect of the normal healing process, mainly resulting from uncontrolled proliferation and migration of arterial smooth muscle cells, as well as excessive expression and deposition of extracellular matrix (ECM) [9,10]. Various compounds targeting inflammation, platelet activation and cell proliferation have been attempted to avoid the neointimal hyperplasia. The highest success showed antiproliferative drugs sirolimus and paclitaxel [11]. Stents coated with permanent polymers containing either of these drugs significantly reduced neointimal hyperplasia through inhibition of smooth-muscle cell proliferation at an early period of stenting [12,13]. On the other hand, concerns about the efficacy and safety of DES have been raised in clinical studies with follow-up beyond the restenosis -period. Published data from meta-analyses and clinical trials comparing DES to BMS suggested higher rates of late clinical events in patients receiving DES [14–17]. The antiproliferative drugs released by DES interfere with the natural vascular healing process by arresting the endothelial cell proliferation and growth on the stent surface [18]. Late (30 days – 1 year) and very late (>1 year) stent thrombosis are the major clinical consequences of impeded endothelial healing in patients receiving DES [19]. While the cause of late and very late thrombosis is recognized to be multifactorial, it can in part be attributed to the polymeric materials used as coatings in DES.

The commonly used DES utilize a permanent coating that remains in the body after complete drug elution. The permanent presence of polymers in the vessel wall is an important factor which can influence local tissue response and alter processes of the wound healing cascade [20]. Consequently, the initial inflammatory reaction to the foreign body may then transform into persistent immune response, limiting the clinical outcome. Indeed, tissues obtained by directional atherectomy six to ten months after DES implantation revealed considerable amounts of T-cells and macrophages and, interestingly, the localized inflammatory reaction was substantially more pronounced compared to that observed in BMS-samples [21]. The persistent inflammation caused by DES, has been proposed to underline the so called catch-up phenomenon, i.e. the occurrence of late stenosis (six month to three years post-implantation), which along with thrombosis, remains an important unresolved issue in the DES era [22–24].

Although stent-related complications in DES are still very rare, their severity necessitates the further improvement of stent technologies [25]. In this context, the use of biodegradable polymers offers an advantage that once drug elution is complete, no non-functional polymer is left behind [26]. Thus, DES with biodegradable polymeric coatings could offer early phase benefits of DES in combination with a remaining permanent stent platform at later stages, decreasing the potential risk for complications.

Despite numerous DES with biodegradable coatings being available commercially, there are still many challenges remaining for novel biodegradable polymers, including the optimal biocompatibility, thrombogenicity and degradation time of the polymer. In particular, biodegradable polyesters, such as poly(D,L-lactide-co-glycolide) (P(DLLA-co-GA) and poly(L-lactide) (PLLA), are used as polymeric coatings for DES [27].

Taking into consideration the accumulating need for developing biodegradable polymeric materials enabling enhanced endothelialization and excellent hemocompatibility, the aim of the present study was to investigate the biodegradable polymers PLLA and poly(D,L-lactide) (PDLLA) and some of their copolymers, such as poly(D,L-lactide-co-glycolide) (P(DLLA-co-GA)), poly(L-lactide-co-glycolide) (P(LLA-co-GA)) and poly(L-lactide-co-ε-caprolactone) (P(LLA-co-CL) by means of their biocompatibility and thrombogenicity, endothelial cell adhesion and viability. In addition, different wet- and plasma-chemical surface modifications were performed to positively influence the bio- and hemocompatibility by generating functional amino and hydroxyl groups.

## Materials and Methods

### Polymer film fabrication

Five different polymers and their surface modified forms were assessed in this study: Resomer L 210 S (poly(L-lactide)), PLLA, intrinsic viscosity in chloroform: 3.8 dL/g), Resomer R 207 S (poly(D,L-lactide)), PDLLA, 1.5 dL/g), Resomer LG 857 S (poly(L-lactide-co-glycolide)), P(LLA-co-GA), 85/15% n/n, 6.0 dL/g), Resomer RG 858 S (poly(D,L-lactide-co-glycolide)), P(DLLA-co-GA), 85/15% n/n, 1.5 dL/g), which were all purchased from Evonik Industries AG (Darmstadt, Germany) and Purasorb PLC 7015 (poly(L-lactide-co-ε-caprolactone)), P(LLA-co-CL), 70/30% n/n, 1.5 dL/g) available from Corbion (Amsterdam, The Netherlands).

All polymer films were prepared using the following procedures: A portion of 1 g polymer was solved in 25 mL of chloroform (p. a., Mallinckrodt Baker), and given into a petri dish (Ø = 9 cm), which was wiped out with acetone. The remaining chloroform was evaporated until an approx. 150 μm thick film remains. A dipping method was preferred for Resomer L210 S to receive a plane polymer film. Therefore, 2.6 g of PLLA was solved in 100 mL of chloroform (p. a., Mallinckrodt Baker). A cylindrical shaped metallic body was immersed into this solution several times until an approx. 100 μm thick film was achieved. Afterwards, the film was removed from the metallic body using a scalpel and flattened out. Films were finally washed for 2 days in methanol (LiChrosolv, Merck KGaA), two days in distilled water (MilliQ Ultrapure, R = 18.2 MΩ.cm at 25°C) and dried for 7 days in a vacuum cabinet drier (model 45001, Bioblock Scientific) at 40°C.

### Polymer surface modification

**Fig 1:**
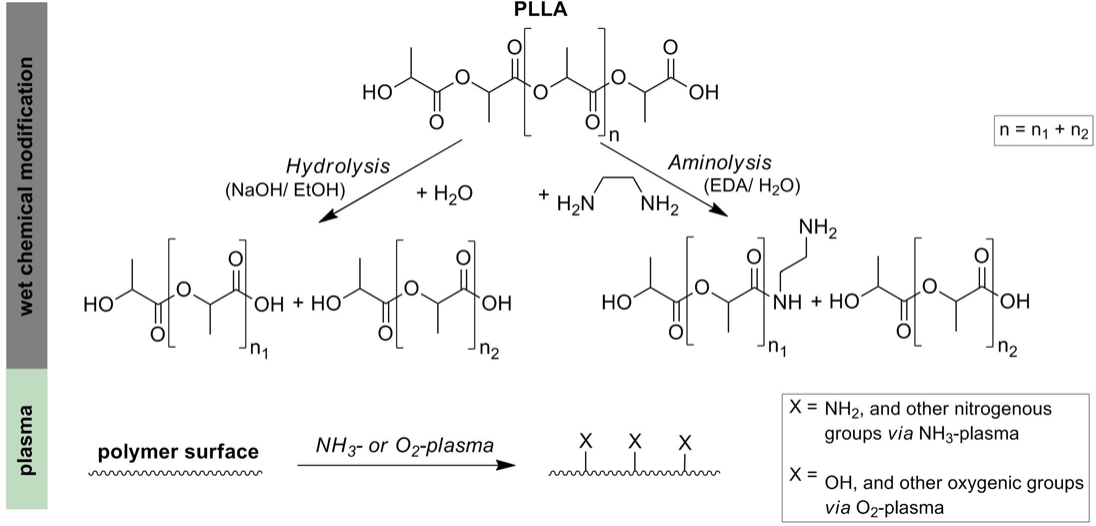
Chemical reactions of the performed wet- and plasma-chemical modifications. Supposed schematic modifications are exemplary demonstrated for PLLA. Wet-chemical polymer surface modifications were performed by alkali hydrolysis (NaOH in ethanol) generating OH groups or by aminolysis (EDA in water) generating NH_2_ groups. Plasma chemical methods were used to generate NH_2_ and other nitrogenous groups (NH_3_-plasma) or OH and other oxygenic groups (O_2_-plasma).

To increase the number of hydroxyl (-OH) groups a wet-chemical degradation of the polymer surfaces under basic conditions was performed. For this, polymers were hydrolyzed for 2 hours at 37°C in a 0.25 M sodium hydroxide (NaOH, p. a., Sigma-Aldrich, St. Louis, USA) solution containing ethanol and water (50/50% v/v) at 37°C according to Yang and colleagues [28] (Fig 1). For generating amino (-NH_2_) groups polymers were immersed for 1 hour in 3 M ethylenediamine (EDA, p. a., Fluka, St. Gallen, Switzerland) at 37°C according to García-García and colleagues [29].

The plasma activation processes were performed via plasma etching (PE) electrode for 10 minutes and 60% generator output in an ammonia (NH_3_) or for 8 minutes and 40% generator output in an oxygen (O_2_) radio frequency (RF) plasma generator (frequency 13.56 MHz, power 100 W, Diener electronic GmbH & Co. KG, Ebhausen, Germany) at a low pressure of 0.3 mbar.

The chemical reactions of the performed wet- and plasma-chemical modifications are summarized in Fig 1.

### Surface characterization

The surface modifications were analyzed by contact angle measurements of sessile drops (water) on the surface using a Contact Angle System (OCA 20, Dataphysics Instruments GmbH, Filderstadt, Germany) and SPSS software 15.0. Mean values and standard deviations were calculated from 10 samples with n = 2 measurements per sample (one top and one back side).

### Cell Culture

Human coronary artery endothelial cells (HCAEC), human umbilical vein endothelial cells (HUVEC) and immortalized murine fibroblast cell line L929 were used. All cells were incubated in a humidified incubator at 37°C with 5% CO_2_ and the culture medium was changed every 48 hours. Upon reaching confluence in the flask, cells were trypsinated and subcultured for the next passage. The cells from the forth to fifth passages were used in these studies.

HCAEC and culture media were purchased from PromoCell (Heidelberg, Germany). The HCAEC were cultured in the endothelial cell growth medium MV + SupplementMix with fetal calf serum (5%), endothelial cell growth supplement (0.04%), recombinant human EGF (10 ng/mL), heparin (22.5 μg/mL) and hydrocortisone (1 μg/mL).

HUVEC were harvested from umbilical cords collected following normal pregnancies after delivery at the Department of Obstetrics and Gynecology, University of Rostock. The study protocol was approved by the Ethics committee of Rostock University Medical Center (approval ID: 38/2004), and written informed consent was obtained from all participants. The cells were isolated through enzymatic digestion by 10-minutes-incubation with 0.05% collagenase at 37°C. This was followed by centrifugation at 50 g (10 minutes).

For expansion, HUVEC were cultured in MCDB131 medium (PAN Biotech) supplemented with 10% fetal calf serum, 0.1 ng/mL EGF, 1 ng/mL bFGF, 5 U/ml heparin, 1 μg/mL hydrocortisone, 0.4% endothelial cell growth supplement, 100 μg/ml penicillin, 100 μg/mL streptomycin and 2 mM L-glutamine.

L929-mouse fibroblasts were cultured in Dulbecco‘s Modified Eagle Medium (DMEM), supplemented with fetal calf serum (10%), 44 mM NaHCO_3_, 25 mM HEPES, 100 U/mL penicillin and 100 μg/mL streptomycin.

For the incubation on polymer surfaces, all three types of cells were seeded onto punched circular discs (Ø = 6 mm) in a concentration of 0,7*10^5^ cells/mL and cultivated under standard culture conditions. Cells grown on native and modified polymers for 72 h were examined using different approaches whereby the cells incubated in cell culture wells served as control.

### Cell viability assay

After the 72 h incubation period on the polymer surfaces, cell viability was determined using the CellQuanti-Blue™ assay (BioAssaySystems, Hayward, CA, USA) according to the manufacturer’s instructions. This assay measures the reduction of the substrate resazurin to resorufin by cellular reductases, giving quantitative information on cell metabolic activity and thus, viability.

Briefly, culture medium was replaced by 10% CellQuanti-Blue reagent diluted in DMEM and incubated for 2 h under standard conditions. Thereafter fluorescence emission was measured at 590 nm with 544 nm excitation using a microtiter plate reader (FLUOstar OPTIMA, BMG Labtech, Offenburg, Germany).

Three independent experiments were performed for each experimental setup. Measurements of one single experiment were performed in six parallels. Cells incubated in microwells without polymers served as control. As a positive control served cells treated with 1*10^−4^ M tetraethylthiuram (TETD, Sigma-Aldrich, Seelze, Germany). All viability values were calculated relative to the control whose viability was set to 100%.

### Confocal laser-scanning microscopy

For confocal microscopy, confluent cell monolayers were fixed in 4% paraformaldehyde (PFA) for 30 min. at room temperature, followed by 3 times rinsing in PBS (pH = 7.4). For immunocytochemistry, samples were incubated overnight with a primary murine monoclonal anti-human CD31 antibody (1:20, DAKO) diluted in DAKO-antibody-diluent. On the next morning, the cells were rinsed again in PBS, and incubated with Alexa Fluor 488 donkey secondary anti-mouse antibody (Life Technologies GmbH, Darmstadt, Germany) used at a dilution (1:100) for one hour at room temperature.

For cytoskeleton staining, the cells were fixed in the same way like for immunocytochemistry. Thereafter 1 hour incubation with Tetramethylrhodamine (TRITC)-conjugated phalloidin (500μg/ml, Sigma-Aldrich, Taufkirchen, Germany) was performed, followed by three times rinses in PBS (pH = 7.4).

Overall, the cell nuclei were counterstained with Hoechst 33342 (1:500, Sigma-Aldrich, Taufkirchen, Germany) for 3 minutes. After final rinses specimens were coverslipped for confocal laser-scanning microscopy (FluoView FV1000; Olympus).

### Scanning electron microscopy

Cell monolayers were fixed with a solution of 25% glutaraldehyde and 0.2M sodium cacodylate in PBS for 30 min and stored at 4°C. After washing with sodium phosphate buffer and short purging with ethanol (30%), samples were dehydrated in graded ethanol (50%, 75%, 90% and 100%). After that the samples were critical-point dried with CO_2_ according to the manual of the device CPD 7501 (Quorum Technologies Ltd, Laughton, Lewes, East Sussex, Great Britain). As the investigated samples are insulating, the sample surface was coated with a thin film of gold. The SEM images were acquired with a scanning electron microscope (Quanta FEG 250, FEI Deutschland GmbH) operating in high vacuum and 10 kV, using an Everhart-Thornley secondary electron detector (ETD).

### Quantification of platelet activation via detection of β-thromboglobulin

Hemocompatibility analysis was performed using whole human blood obtained from healthy volunteers using Diacan 17 gauge-needle (1,5x20 mm) butterfly devices with polyvinyl chloride tubing (B.Braun, Melsungen, Germany) at the Department of Transfusion Medicine of Rostock University. The blood was slowly dropped into open Na-citrate tubes (10 mL S-Monovetten® mit 1 mL Na-Citrat [0.11 mol/L], Sarstedt, Nümbrecht). Immediately after blood collection the samples were centrifuged for 20 min at room temperature and 800 rpm (110 g) in a universal 32 R centrifuge (Hettich, Tuttlingen, Germany) and the supernatant platelet-rich plasma was separated. One mL of supernatant was transferred into CTAD tubes (citrate, theophylline, adenosine, dipyridamole- tubes, Sarstedt, Nümbrecht, Germany) and stored cold, serving as a control (NC_β-TG 0_). For the remaining supernatants, 1 mL were incubated for 30 minutes at 37°C (incubator B 290, Heraeus, Hanau, Germany) in contact to punches (ø = 10 mm) of native and modified polymers as well as a second control (NC_β-TG 30_), which was treated as a sample, but without specimen contact, and then transferred to CTAD tubes. For the positive control, samples were treated with 10 μL of thrombin stock solution (100 Units/mL in a 0.1% (w/v) BSA solution), (thrombin from human plasma, Sigma-Aldrich, Saint Louis, MO, USA). Thereafter, the tubes were centrifuged for 30 min at 4000 rpm (2000 g) and 2°C in a pre-cooled centrifuge (Laborfuge 400R, Heraeus, Hanau, Germany) and after that, about one third of the plasma supernatant from the middle part of the tube was taken and 1:21 diluted with the dilution buffer of the β-TG ELISA kit (ASSERACHROM® β-TG-ELISA, Diagnostika Stago S.A.S., Asnières sur Seine, France). 200 μL of these diluted samples were incubated on strips coated with anti-human β-TG-rabbit immunoglobulin G. Thereafter, the immunoenzymatic determination of β-TG was performed according to the manufacturer's instructions. Using a microtiter plate reader (FLUOstar OPTIMA, BMG Labtech, Offenburg, Germany) the extinction of the samples was measured at a wavelength of 450 nm. Platelet activation was measured by detection of β-thromboglobulin. A polynomial regression curve was plotted out of the measured values of the dilutions of the supplied standard. β-TG concentration (IU/mL) was calculated and shown graphically for every native and modified polymer as well as for the controls.

### Statistical analysis

Statistical analyses were performed using GraphPad Prism 5 data analysis software (GraphPad Software, La Jolla, CA, USA). Statistical differences between unmodified polymers and corresponding modifications were analyzed with one-way ANOVA. Between-group differences were considered to be statistically significant for p-values ≤ 0.05 (p*≤ 0.05, p**≤ 0.01, p***≤ 0.001).

## Results

### Surface characterization

**Fig 2:**
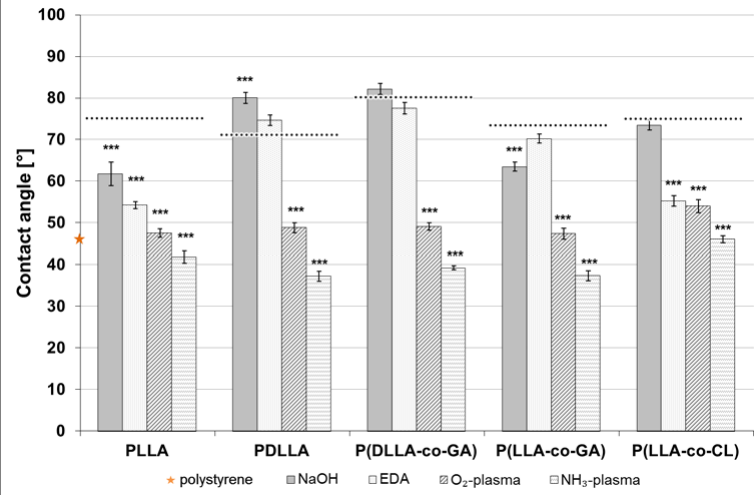
Contact angle measurements. Mean values of contact angle measurements of water by sessile drop method on unmodified and modified polymer surfaces (n = 10) ± SEM. Dotted lines indicate the contact angle of unmodified polymers. (p*≤ 0.05, p**≤ 0.01, p***≤ 0.001).

For confirmation of surface modification, all polymer films were examined by contact angle measurement. Thereby, plasma activation and wet-chemical modifications were compared with unmodified polymer surfaces ensuring improvements in wettability. The lower the water contact angle, the higher is the wettability or hydrophilicity of the material. As shown in Fig 2, polystyrene exhibited a lower contact angle when compared to all examined polymers in unmodified state. Plasma treatment resulted in strong decreases of contact angles by up to 50%. Overall, the NH_3_-plasma modification exhibited 6° to 11° lower values compared to O_2_-plasma modification. However, both plasma activations decreased the contact angle significantly for all polymers. In contrast, wet-chemical modifications did not affect the contact angle in the majority of cases. Especially after EDA-modification no significant affect in contact angle could be detected on three of five polymers, PDLLA, P(DLLA-co-GA), and P(LL-co-GA). NaOH-modification even increased the contact angle, as shown in Fig 2 for PDLLA.

### Cell viability

**Fig 3:**
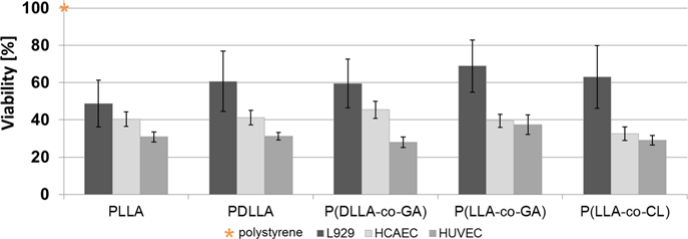
Cell viability measurements of three different cell types. Relative cell viability after 72 h growth on unmodified polymer surfaces, n = 6 (for each cell type). Orange star indicates the viability of cells on polystyrene (control), which was set to 100% (asteric). Data are presented as mean of three independent experiments ± SEM.

The viability of cultured L929 fibroblasts and endothelial cells HCAEC and HUVEC after cultivation on the different unmodified polymer surfaces was measured by CQB-assay. Cell viability on reference material polystyrene (control) was set to 100% (Fig 3, asteric). After 72 h incubation on different unmodified polymer surfaces all three types of cells exhibited lower viability compared to the NC (Fig 3).

Among the cell types, the highest viability on the respective polymers was observed for L929 fibroblasts, followed by HCAEC. On every polymer examined, HUVEC exhibited the lowest relative viability, varying between 28 and 37% of the NC (Fig 3).

**Fig 4:**
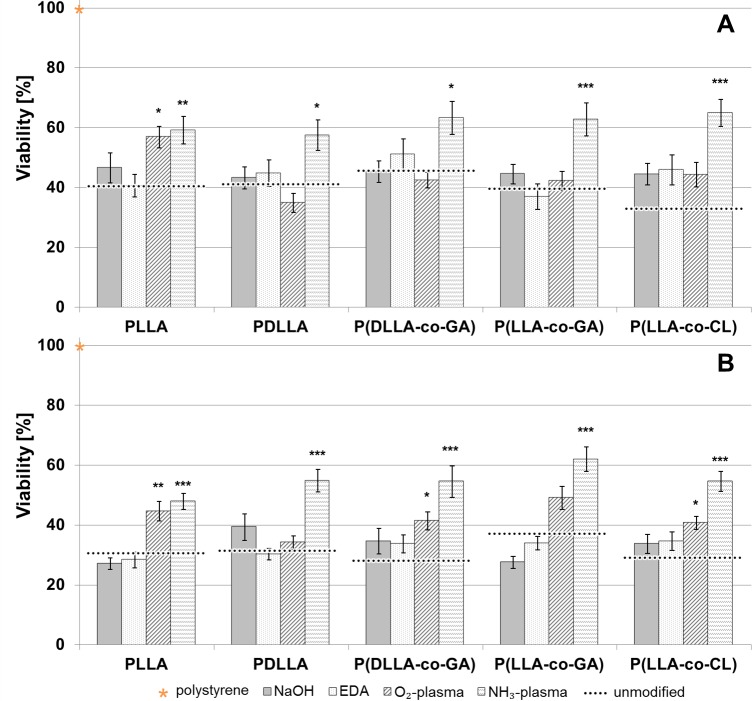
Cell viability measurements of HCAEC and HUVEC grown on unmodified and modified polymer surfaces. Relative cell viability of HCAEC (A) and HUVEC (B) after 72 h incubation on the polymer surfaces activated by means of wet- and plasma-modification, n = 6 (for each cell type). Dotted lines indicate the relative viability of respective unmodified polymers. The viability of polystyrene was set to 100% (asteric). Data are presented as mean of three independent experiments ± SEM. (p*≤ 0.05, p**≤ 0.01, p***≤ 0.001).

As shown in Fig 4, all four different wet- or plasma-chemical surface modifications influenced the viability of both, HCAEC (Fig 4A) and HUVEC (Fig 4B) when compared to that on unmodified ones (black dotted lines). The most pronounced positive effect was seen in case of NH_3_-plasma modification, which yielded the highest viability values for both cell types on every polymer surface tested (Fig 4A and 4B). In all cases, the difference of viability following NH_3_-plasma modification was statistically significant, compared to unmodified polymers.

The effect of the three other modifications (O_2_-plasma, NaOH and EDA) varied between polymers and cell types. In majority of cases, the differences between unmodified and modified states did not reach significance. As for HCAEC (Fig 4A) the only statistically significant improvement was seen for PLLA upon O_2_-plasma modification.

In case of HUVEC (Fig 4B), statistically significant improvement of biocompatibility could be reached for PLLA, P(DLLA-co-GA) and P(LLA-co-CL) upon O_2_-plasma modification. In contrast, wet-chemical modifications with NaOH- and EDA did not influence any of examined polymers significantly.

### Cell adhesion and morphology

**Fig 5:**
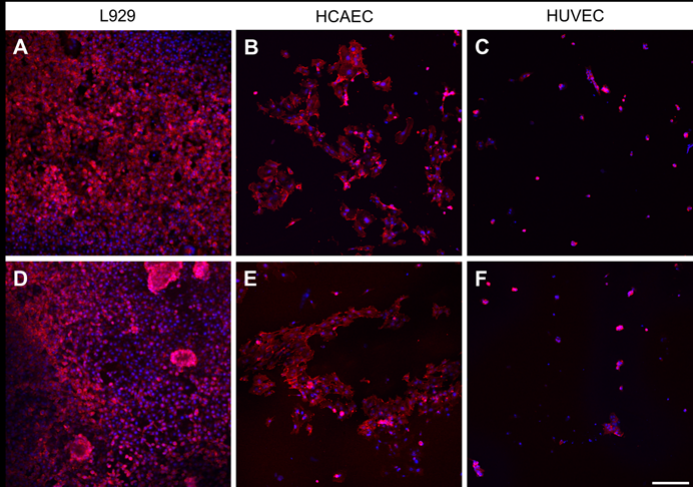
Confocal images of cells grown on two different polymers. Actin filaments are seen in red (phalloidin-staining), cell nuclei in blue (Hoechst-staining) The upper row demonstrates the cell adhesion of L929 (A), HCAEC (B) and HUVEC (C) after incubation on unmodified P(LLA-co-GA).The lower row demonstrates the adhesion of the same cells, L929 (D), HCAEC (E) and HUVEC (F) following incubation on unmodified P(LLA-co-CL). Scale bar = 200 μm.

The adherence to the unmodified polymeric substrates was compared between the three cell types. For all polymers, the phalloidin staining revealed a L929 cell adhesion superior to that of HCAEC and HUVEC, which was in agreement with quantitative data obtained with viability assay. Fig 5 demonstrates representative confocal images of cells grown on two different polymers, P(LLA-co-GA) and P(LLA-co-CL). A very good adhesion to unmodified P(LLA-co-GA) was clearly seen for L929 (Fig 5A), followed by HCAEC (Fig 5B), whereas the HUVEC demonstrated only a poor adherence to the same substrate (Fig 5C). Also the second polymer, P(LLA-co-CL),demonstrated the same tendency regarding cell adhesion (Fig 5D through 5F). Also here, the L929 adhered at highest amount (Fig 5D), followed by HCAEC (Fig 5E), whereas only some occasional HUVEC could be seen on the same material (Fig 5F).

**Fig 6:**
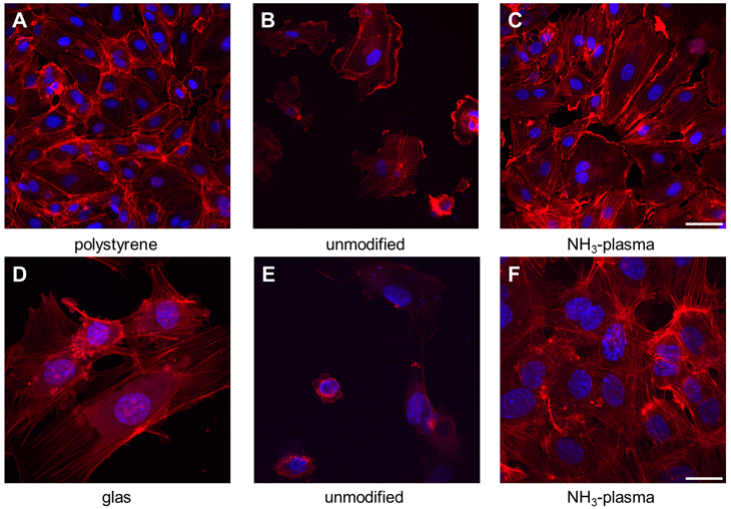
Representative micrographs of HUVEC grown on polystyrene and two different unmodified and modified polymers. Confocal micrographs of actin-stained HUVEC (red staining) grown on polystyrene (A), glass (D) and unmodified polymers P(DLLA-co-GA) (B) and PLLA (E). A better cell adhesion and actin filament on NH_3_-activated P(DLLA-co-GA) (C) and PLLA (F), when compared to the respective polymers in unmodified state ((B) and (E), respectively). Overall, the blue staining (Hoechst-staining) indicates cell nuclei. Scale bars = 50 μm in (A), (B), (C) and 20 μm in (D), (E) (F).

In the next step it was examined whether the surface modification of polymers can promote endothelial cell adhesion. In agreement with CQB-assay, also here, the NH_3_-plasma-activation was the only modification, which univocally influenced the adhesion of HCAEC and HUVEC on all five polymers. Fig 6 demonstrates the representative micrographs of HUVEC grown on two different polymers P(DLLA-co-GA) and PLLA in unmodified state (Fig 6B and 6E, for P(DLLA-co-GA) and PLLA, respectively) and following NH_3_-plasma-activation (Fig 6C and 6F, for P(DLLA-co-GA) and PLLA, respectively). On the polystyrene (Fig 6A), as well as on the culture glass (Fig 6D), the cells exhibited an intense staining with noticeable organization of thin actin filaments stretching through the entire length of the cells. Only a few HUVEC tended to attach to unmodified polymers, and even those revealed only a diffuse background staining and an actin arrangement could barely be seen (Fig 6B and 6E). The NH_3_-activation resulted not only in an increase of cell adhesion (Fig 6C and 6F), but also in staining intensification and actin filament organization comparable to that seen in control (Fig 6A and 6D).

**Fig 7:**
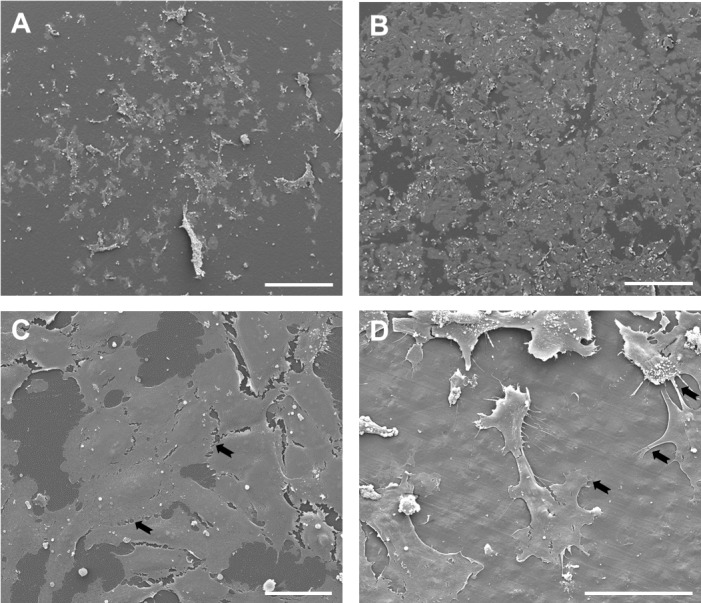
Representative SEM micrographs demonstrating the behavior of HUVEC on unmodified and modified PLLA. SEM micrographs of HUVEC grown on unmodified (A) and NH_3_-modified (B) PLLA. (C) and (D) show details of cell morphology and distribution on NH_3_-modified PLLA at a higher magnification. Black arrows in (C) indicate zipper-like manner cell contacts. In (D) extensions of the plasma membrane are marked with black arrows. Scale bars = 500 μm in (A) and (B), 50 μm in (C) and (D).

These findings on cell adhesion and spreading could be further confirmed using scanning electron microscopy (SEM). An example demonstrating the behavior of HUVEC on unmodified and modified polymer is shown for PLLA (Fig 7). As opposed to the unmodified PLLA with only sparse cell distribution (Fig 7A), a confluent cell monolayer could be visualized on the NH_3_-activated PLLA (Fig 7B). The higher magnification revealed flattened cells with normal shape and form attached to the surface (Fig 7C). In some cells contacts with neighboring cells through short cell processes in a zipper-like manner could be visualized (Fig 7C, black arrows). At a higher magnification, fine extensions of the plasma membrane, filopodia, attaching the substrate could be demonstrated in adherent cells (Fig 7D, black arrows). Similar SEM-findings were seen in case of other four polymers, revealing that the NH_3_-plasma surface modification had the greatest impact on cell adhesion and ability to bind to substrate and adjacent cells (not shown).

**Fig 8:**
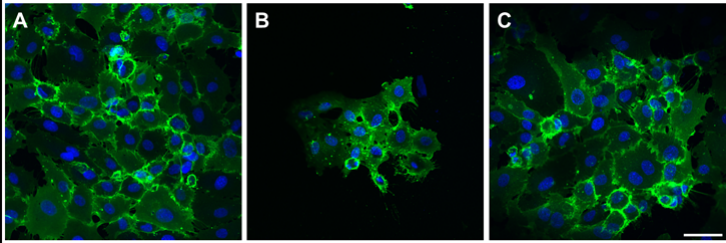
Confocal micrographs of HUVEC grown on polystyrene and on representative unmodified and modified P(DLLA-co-GA). Stained with anti CD-31 (green). (A) HUVEC grown on polystyrene, (B) HUVEC grown on unmodified P(DLLA-co-GA), (C) staining patterns of HUVEC, grown on NH_3_-plasma modified P(DLLA-co-GA) are comparable to those in control (A). The blue staining (Hoechst-staining) indicates cell nuclei. Scale bar = 50 μm.

CD-31 immunocytochemistry was used as a positive indicator of successful formation of cell–cell junctions. Fig 8 shows examples of CD-31 stained HUVEC on polystyrene (A), unmodified model polymer P(DLLA-co-GA) (B) and on the same polymer surface after NH_3_-plasma modification (C). In contrast to very few HUVEC detected on unmodified P(DLLA-co-GA) (Fig 8B), a confluent cell monolayer was visualized on polystyrene, with a faint homogeneous background CD-31-staining in cytoplasma, and more intense localization of the glycoprotein in cell-to-cell contact sites (Fig 8A). After surface modification, the P(DLLA-co-GA) seemed to attract a much higher number of HUVEC (Fig 8C). Notably, the cell on the modified polymer exhibited a healthy morphology and CD-31 staining pattern (Fig 8C) similar to that observed in HUVEC on polystyrene (Fig 8A).

### Platelet activation

**Fig 9:**
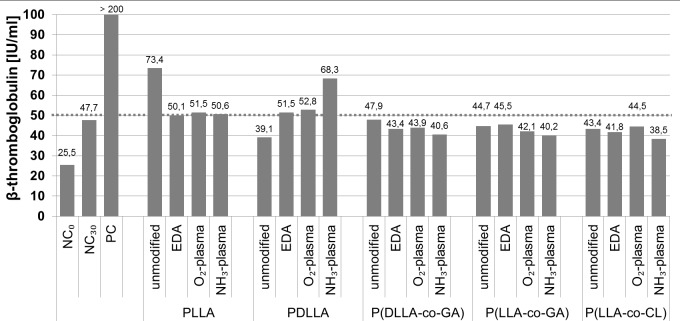
Plasma β-thromboglobulin concentrations after incubation with unmodified and modified polymers. The dotted line represents the upper limit of normal β-thromboglobulin concentration. NC = negative control, PC = positive control.

To evaluate the thrombogenetic potential of the polymers and the possible influence of different wet- and plasma- chemical modifications we measured levels of β-thromboglobulin in blood plasma after incubation with polymer samples (Fig 9). β-thromboglobulin is a protein exerted from platelet granules upon activation. According to the literature, 50 IU/mL is the upper plasma limit of β-thromboglobulin [30]. In unmodified state, only PLLA was shown to activate platelets as indicated by elevated levels of β-thromboglobulin (73.4 IU/mL). However, all investigated modifications improved the thrombogenicity of PLLA, reducing the protein level to the normal. The other four polymers did not exhibit thrombogenic potential, and β-thromboglobulin concentration in plasma after incubation with polymer samples (unmodified and modified) corresponded to the normal values (Fig 9). Only the NH_3_-plasma modified PDLLA elevated the level of β-thromboglobulin to 68.3 IU/ml.

## Discussion

Despite all the benefits from first-generation DES questions arose on the long-term safety of these devices, especially in relation to late and very late thrombosis [31–33]. Another major drawback of DES is restenosis occurring due to neointimal tissue proliferation after complete release of antiproliferative drugs at later stages after stenting [34].

Focusing on the major limitations of current DES, recent research has included modifications to the used drugs and polymer coatings [35]. In this context, biodegradable polymer coatings and scaffolds based on bioabsorbable polymer materials have been developed (Table 1).

**Table 1:** Biodegradable polymers in stent technologies.

Name	Polymer material	Polymer function
Absorb BVS	PLLA, PDLLA	scaffold, coating
BioMatrix FLEX^TM^	PLA	coating
DESyne	PLLA	coating
DESolve	PLLA	scaffold
euca TAX	PLGA	coating
Fantom scaffold family	Desaminotyrosine polycarbonate	scaffold
FORTITUDE	PLLA	scaffold
IDEAL	Polyanhydride ester	scaffold
Igaki-Tamai	PLLA	scaffold
JACTAX®	DLPLA	coating
MeRes^TM^	PLLA	scaffold
NEVO^TM^	PLGA	coating
Nobori	PLA	coating
RESORB, ReZolve, ReZolve2	Tyrosine polycarbonate	scaffold
Synergy®	PLGA	coating
XINSORB	PLLA, PLLA/PDLLA	scaffold, coating
Yukon® Choice PC	PLA	coating

PLA: polylactic acid, PLGA: polyglycolide and poly(D,L-lactic-co-glycolic acid), DLPLA: D-lactic poly(lactic acid), PLLA: poly-L-lactic acid; PDLA: poly(d-lactic acid).

Polylactides PLLA or PDLLA are the most common biodegradable polymer materials used for scaffolds [36].

While favored for sustained drug release and degradability, these polymers still need further research concerning their biocompatibility and hemocompatibility. PLLA has demonstrated a very low affinity for endothelial cells and also its thrombogenicity remains controversial [37].

In the present study we applied different surface modification approaches in attempt to improve biocompatibility and haemocompatibility of biodegradable polymers.

In unmodified state, all polymers demonstrated relative poor biocompatibility which was considerably improved upon plasma and wet-chemical modifications. Overall, the superiority of plasma modification over wet-chemical treatment was obvious.

Previously, oxygen, hydrogen and nitrogen plasma modifications have been successfully applied to durable polymers such as PVA (poly (vinylalcohol)), PDSM (polydimethylsiloxane/silicone) and POSS (polyhedral oligomeric silsesquioxane poly(carbonate-urea)), which are widely used in cardiovascular surgery [38–41] In contrast, only little work has been done to modify the biodegradable polymers in this way. To our knowledge surface activation with plasma has been done so far only for PCL (Poly-ε-caprolacton) by creation of amino groups [42], for PLLA by creation of carboxyl groups [37], and for PLGA (poly(L-lactide-co-glycolide)) by creation of hydroxyl groups [43]. In our study, significant improvement of endothelial cell viability and adhesion could be reached during NH_3_-and O_2_-plasma treatment, and this was true for all five biodegradable polymers. As shown by immunocytochemistry and SEM, not only the cells better survived on the modified surfaces, but also exhibited healthy cytoskeleton, very good intracellular and focal contacts, comparable to control cultures After exposure to NH_3_-or O_2_-plasma, nitrogen or oxygen containing groups are introduced on the surface of a polymer [44]. Previously, it has been demonstrated that the number of amino groups on the PLLA surface was only negligible following NH_3_-plasma treatment [45]. The low number of amino groups was attributed to the well-recognized low plasma penetration depth of only 10 nm [46]. A strong decrease in water contact angle accompanied with enhanced wettability was documented for PLLA after 30 seconds of O_2_ and helium plasma treatment, even though the authors were not able to determine functional groups incorporated at the surface [47]. Similarly, only a relatively low number of functional groups could be detected on the surface of PCL, following NH_3_- or O_2_- plasma treatments, which, however, yielded a significant decrease of contact angle suggesting increased hydrophilicity following plasma treatment [42]. Also, in our study the better biocompatibility of NH_3_- or O_2_-plasma-treated polymers results from the enhanced wettability of the polymer surface, as shown by the decrease in contact angle. A literature search revealed that not only endothelial, but also neural and epithelial cells exhibited a substantially improved cell attachment and growth on polymer surface after plasma modification [48,49].

The major drawback of plasma-treatment is that plasma-induced surface characteristics are not permanent and the polymer surfaces incline to recover to their untreated state, limiting their clinical application. Despite different approaches being tested in the last years to overcome this shortcoming [50], it still remains unsolved. On the other hand, plasma modification can successfully be used for covalent coupling of ECM proteins on the polymer surfaces. ECM molecules, enhancing endothelial cell adhesion and proliferation, would not withstand physiological shear stresses when employed as passive surface coatings, necessitating a stronger covalent bonding. While the direct protein coupling to polymers is limited due to their inherent hydrophobicity, the more hydrophilic plasma modified surfaces provide improved conjugation options for biomolecules [37].

Hemocompatibility analyses performed in our study were based on measurements of ß-thromboglobulin concentration and aimed to determine the achieved surface alterations regarding platelet reaction. Platelet activation upon interaction with different surfaces is a major aspect of material hemocompatibility as it could lead to thrombotic complications under *in vivo* conditions [51]. Following activation, platelets release their pro-coagulant granules that contain proteins including ß-thromboglobulin, platelet basic protein, platelet factor-4 and some others, which are released into the plasma, where they can be used as a differential marker for activation. Among all tested polymers, only PLLA exhibited thrombogenic properties in its unmodified state. Previously, the surface of PLLA has been reported to induce platelet and leukocyte activation [52]. Importantly, we could show here, that all performed surface modifications led to the reduction of PLLA thrombogenicity. Within this context, the wet-chemical modification with ethylenediamine (EDA) can be of a particular clinical relevance, due to longer shelf-life compared to plasma treatment.

In conclusion, our study demonstrates for the first time the feasibility of surface modifications via NH_3_- or O_2_- plasma treatments for the biodegradable polylactides PLLA and PDLLA as well as their copolymers. Compared to untreated surfaces, an improved behavior of endothelial cells on polymers could be attained after surface activation with plasma. Furthermore, both wet- and plasma-chemical treatment significantly improved the hemocompatibility of PLLA, P(DLLA-co-GA), P(LLA-co-GA) and P(LLA-co-CL). Studies are underway to further accelerate the re-endothelialization process on plasma-modified polymer surfaces by covalent coupling of growth factors, such as vascular endothelial growth factor or basic fibroblasts growth factor.

## Supporting Information

S1 Data Figshare DOIs. (DOCX)
