# Supplementary material for: Surface Modification of Biodegradable Polymers towards Better Biocompatibility and Lower Thrombogenicity
Source: PLoS One. 2015 Dec 7;10(12):e0142075. doi: 10.1371/journal.pone.0142075 (PMC4671536; doi:10.1371/journal.pone.0142075)
Supplement: S1 Data — (DOCX) [file pone.0142075.s001.docx]

**Figshare DOI**

Fig 1. <http://dx.doi.org/10.6084/m9.figshare.1598119> 
Fig 2. <http://dx.doi.org/10.6084/m9.figshare.1599744> 
Fig 3. <http://dx.doi.org/10.6084/m9.figshare.1599743> 
Fig 4. <http://dx.doi.org/10.6084/m9.figshare.1599745> 
Fig 5. <http://dx.doi.org/10.6084/m9.figshare.1599749> 
Fig 6. <http://dx.doi.org/10.6084/m9.figshare.1599748> 
Fig 7. <http://dx.doi.org/10.6084/m9.figshare.1599746> 
Fig 8. <http://dx.doi.org/10.6084/m9.figshare.1599747> 
Fig 9. <http://dx.doi.org/10.6084/m9.figshare.1599750>
